# Ventricular arrhythmia and sudden cardiac death in hypertrophic cardiomyopathy: From bench to bedside

**DOI:** 10.3389/fcvm.2022.949294

**Published:** 2022-08-18

**Authors:** Hua Shen, Shi-Yong Dong, Ming-Shi Ren, Rong Wang

**Affiliations:** ^1^Division of Adult Cardiac Surgery, Department of Cardiovascular Medicine, The Sixth Medical Center, Chinese PLA General Hospital, Beijing, China; ^2^Department of Cardiovascular Surgery, The First Medical Center, Chinese PLA General Hospital, Beijing, China; ^3^Graduate School, Chinese PLA General Hospital & Chinese PLA Medical School, Beijing, China

**Keywords:** hypertrophic cardiomyopathy (HCM), sudden cardiac death (SCD), ion channel, reentry, calcium homeostasis, sarcomere, gene screening

## Abstract

Patients with hypertrophic cardiomyopathy (HCM) mostly experience minimal symptoms throughout their lifetime, and some individuals have an increased risk of ventricular arrhythmias and sudden cardiac death (SCD). How to identify patients with a higher risk of ventricular arrythmias and SCD is the priority in HCM research. The American College of Cardiology/American Heart Association (ACC/AHA) and the European Society of Cardiology (ESC) both recommend the use of risk algorithms to identify patients at high risk of ventricular arrhythmias, to be selected for implantation of implantable cardioverters/defibrillators (ICDs) for primary prevention of SCD, although major discrepancies exist. The present SCD risk scoring systems cannot accurately identify early-stage HCM patients with modest structural remodeling and mild disease manifestations. Unfortunately, SCD events could occur in young asymptomatic HCM patients and even as initial symptoms, prompting the determination of new risk factors for SCD. This review summarizes the studies based on patients' surgical specimens, transgenic animals, and patient-derived induced pluripotent stem cells (hiPSCs) to explore the possible molecular mechanism of ventricular arrhythmia and SCD. Ion channel remodeling, Ca2+ homeostasis abnormalities, and increased myofilament Ca2+ sensitivity may contribute to changes in action potential duration (APD), reentry circuit formation, and trigger activities, such as early aferdepolarization (EAD) or delayed afterdepolarization (DAD), leading to ventricular arrhythmia in HCM. Besides the ICD implantation, novel drugs represented by the late sodium current channel inhibitor and myosin inhibitor also shed light on the prevention of HCM-related arrhythmias. The ideal prevention strategy of SCD in early-stage HCM patients needs to be combined with gene screening, hiPSC-CM testing, machine learning, and advanced ECG studies, thus achieving individualized SCD prevention.

## Hypertrophic cardiomyopathy: A brief overview

Hypertrophic cardiomyopathy (HCM) is an inheritable disease characterized by ventricular hypertrophy due to either sarcomere protein-coding genes, sarcomere protein-related gene variations, or unknown etiology, in the absence of obvious pathologies or defects such as hypertension or aortic stenosis. The prevalence rate in the general population is 1:200 ~ 1:500 (1). HCM presents a heterogeneous clinical course and phenotypic expression. Patients mostly experience minimal symptoms throughout their lifetime, and some individuals have an increased risk of ventricular arrhythmias and sudden cardiac death (SCD).

### Genetic mutations in HCM

**Figure 1:**
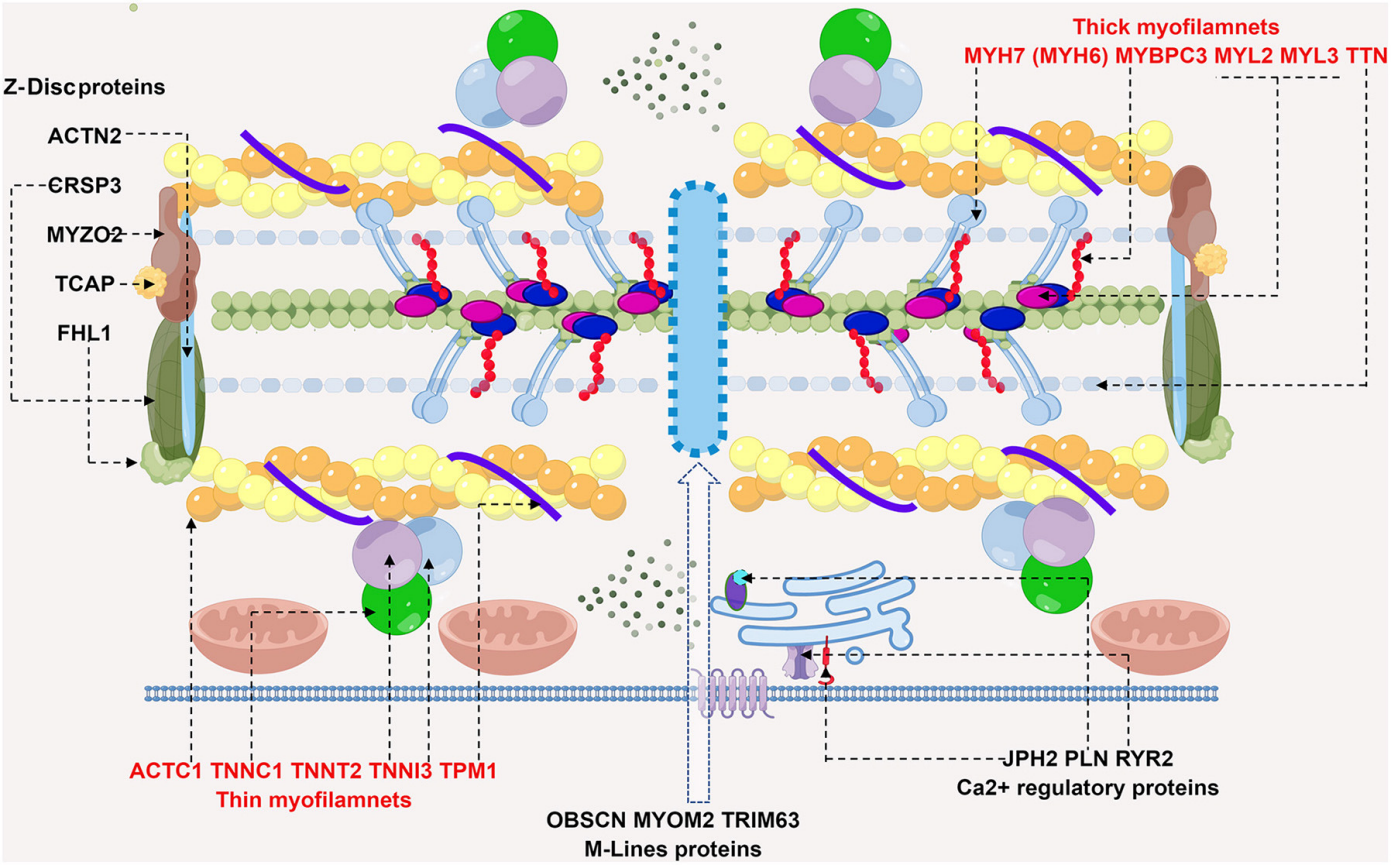
Schematic diagram of potential sarcomere gene mutations involved in HCM. HCM is mainly a disease of sarcomere proteins, which are comprised of thick and thin filaments, Z-Disc, M-lines and calcium regulatory proteins. Shown in the diagram are recognized genes affected in HCM, dashed arrows point to the corresponding proteins. Thick filaments are comprised of MYH7 (myosin heavy chain 7) and MYBPC3 (myosin binding protein C3), MYL2/3 (myosin light chain 2/3) and TTN (titin). Gene screening in HCM patients reach to mutations in MYBPC3 and MYH7, most commonly. ACTC1 (cardiac α-actin 1), TNNT2 (cardiac troponin T), TNNI3 (cardiac troponin I), TNNC1 (cardiac troponin C), and TPM1 (α-tropomyosin) collectively constitute thin filaments. Rare causes of HCM by gene mutations in Z-disc, M line proteins and calcium regulatory proteins are also depicted. Abbreviations: ACTN2, α-actinin 2; CSRP3, cysteine and glycine rich protein 3; MYOZ2, myozenin 2; TCAP, TTN capping protein; FHL1, four and half LIM domain1; OBSCN, obscurin; MYOM2, myomesin 2; TRIM63, tripartite motif containing 63; PLN, phospholamban; JPH2, junctophilin 2; RyR2, type 2ryanodine receptor.

HCM, considered a familial disease, is usually inherited as a Mendelian autosomal dominant trait with variable penetrance (1). More than 2000 variants in over 20 genes encoding myocardial or adjacent Z-disc components have been identified in the pathogenesis of HCM (2) (Figure 1), including the following eight genes with high-level pathogenic evidence: cardiac troponin T (TNNT2), cardiac troponin I (TNNI3), myosin regulated light chain (MYL2), myosin essential light chain (MYL3), cardiac myosin binding protein C (MYBPC3), cardiac muscle β Myosin heavy chain (MYH7), α Cardiac actin (ACTC1), and tropomyosin 1 (TPM1). There are also other genes with moderate evidence to support the pathogenicity of HCM, including cardiac troponin C (TNNC1), junctophilin 2 (JPH2), and cysteine and glycine-rich protein 3 (CSRP3) (3). Among the above-mentioned genes, the MYBPC3 mutation is the most common, accounting for up to 50% of identified mutations, followed by the MYH7 mutation which occurs in 25–40% of patients. Up to 5% of patients have multiple mutations; however, most patients do not carry sarcomere gene variants. The genome-wide association study (GWAS) by Hugh Watkins et al. indicated that a substantial proportion of HCM risk was attributable to the additive effects of common single-nucleotide polymorphism (SNP), especially for sarcomere-negative HCM, and also polygenic common variants partially influenced phenotypic severity in sarcomere variant carriers (4).

### Gene screening in HCM

Gene screening for HCM probands or suspicious families is used to identify relatives with sarcomere gene mutations (genotype positive) but with rare clinical symptoms and imaging evidence (clinical phenotype negative), which is also called preclinical HCM. It is necessary to dynamically evaluate its clinical phenotypic changes. In addition, gene screening effectively determined other metabolic cardiomyopathies related to ventricular hypertrophy phenotypes, such as glycogen storage disorder (Danon disease), the non-catalytic subunit activated by protein kinase adenosine monophosphate γ2 (PRKAG2 cardiomyopathy), lysosomal storage disorder (Fabry disease), cardiac amyloidosis and other congenital metabolic diseases (5).

Despite progress in cardiac genetics and the introduction of next-generation sequencing technology, approximately 40% of HCM cases are still unexplained, suggesting that there may be oligogenic or polygenic mechanisms. The incomplete penetrance of gene mutations and the inherent variability of disease performance complicate the genotype and phenotype correlation in HCM (3). Hugh Watkins et al. showed that polygenic risk and modifiable risk factor such as diastolic blood pressure (DBP) substantially influenced sarcomere-negative patients with HCM and their relatives (4).

## Ventricular arrhythmias and SCD in HCM

### Epidemiology of ventricular arrhythmias and SCD

HCM patients are prone to arrhythmias, including atrial fibrillation, simple ventricular premature beats (VPB), non-sustained ventricular tachycardia (NSVT), sustained ventricular tachycardia (SVT), ventricular fibrillation (VF), hemodynamic failure and sudden cardiac death (SCD) (6). SCD, the most devastating complication of HCM, may be the first manifestation in some patients. The incidence of SCD in HCM patients is 0.5–1% per year, mainly affecting children and young people <30 years old. Also, SCD risk decreases with age, and this adverse outcome is rare in HCM patients over 60 years old. The cumulative incidence of SCD in childhood HCM patients over 5 years is approximately 8–10%, leading to the recognition of HCM as the most common cause of SCD in young people in North America (6, 7).

### Debate on SCD risk stratification

Previous studies focused on the exploration of risk factors for SCD found that the pathophysiological mechanisms of ventricular arrhythmia and SCD, including genotyping, extensive myocyte disarray, abnormalities of the intramural microvasculature, and interstitial fibrosis, were complex. This brought considerable complexity to the establishment of the risk stratification model (8). For example, intramural microvascular dysfunction leads to repetitive episodes of ischemia and thus resulting in myocyte death and fibrotic replacement (9), which was regarded as the ischemic precursor of fibrosis and predominates at the earliest stages (10). However, in younger patients, recently ischemia was found to strongly correlated with LGE in CMR, at least in part, explained the heterogeneity in the extent of fibrosis found among younger patients, indicating the importance of targeted therapies with significant impact on the disease natural history and prognosis (11).

**Figure 2:**
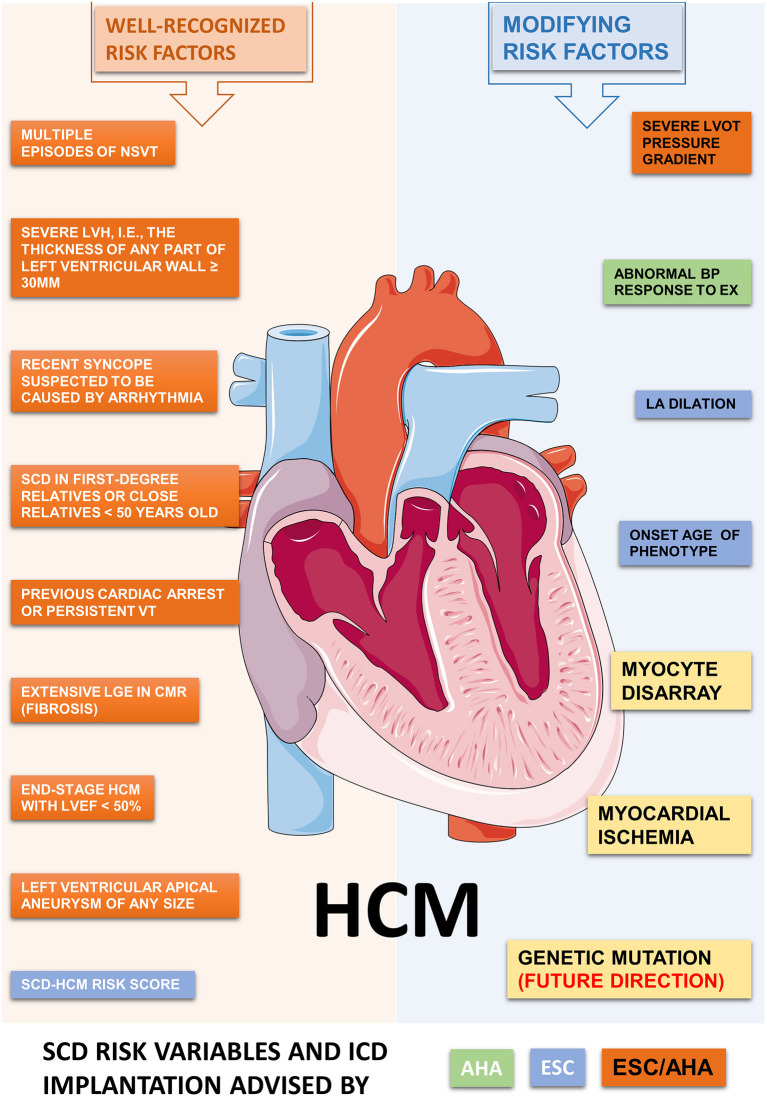
Potential risk factors contributed to ventricular arrhythmia and SCD in HCM. The ACC/AHA and ESC shared a consensus on the recommendations for ICD implantation in HCM at 2003. However, major differences have aroused recently on the SCD risk classification and indications for ICD implantation. We summarize the well-recognized and some modifying risk factors related to SCD in HCM, as recommended by ACC/AHA and/or ESC guidelines. Abbreviations: HCM, hypertrophic cardiomyopathy; VT, ventricular tachycardia; ICD, implantable cardiac defibrillator; SCD, sudden cardiac death; LVH, left ventricular hypertrophy; NSVT, non-sustained ventricular tachycardia; BP, blood pressure; LGE, late gadolinium enhancement; LVOT, left ventricular outflow tract; LV, left ventricle; EF, ejection fraction; EX, exercise; LA, left atrium.

At present, the well-recognized risk factors for SCD include a previous cardiac arrest or persistent ventricular arrhythmia, SCD in first-degree relatives or close relatives <50 years old, recent syncope suspected to be caused by arrhythmias, severe LVH (a thickness of any part of the left ventricular wall ≥30 mm), left ventricular apical aneurysm of any size, and end-stage HCM with a left ventricular ejection fraction (LVEF) of < 50% (12, 13). Unfortunately, SCD episodes could occur in patients without the conventional risk factors mentioned above, leading to the further exploration of novel SCD risk variables. Risk modifying factors include genetic mutations, LGE in CMR, patient age, multiple episodes of NSVT, myocardial ischemia, LVOT pressure gradients, etc. (14) (Figure 2).

The recommendations of the AHA (15) and the European Society of Cardiology (ESC) guidelines (16) for the primary prevention of HCM with ICDs are inconsistent and sometimes, even contradictory. Real-world decision-making data about ICD implantation from North American and European experts work together to improve risk stratification strategies (13). Prophylactic ICDs for high-SCD-risk patients have reduced the disease-related mortality by more than ten times, to attain a mortality rate of 0.5% per year. The five-year and ten-year survival rates (confined to HCM deaths) were 98 and 94%, respectively, which are not different from the expected all-cause mortality rate in the general U.S. population (17).

However, the existing SCD risk stratification models have apparent limitations (8). Although the risk-scoring algorithms effectively identify high-risk patients in the late stage of disease progression, they have limited accuracy in identifying patients with rare structural remodeling and mild disease manifestations (12). Many young asymptomatic patients, even without advanced structural LV remodeling, fibrosis, and severe microvascular dysfunction, suffer from ventricular arrhythmias and SCD. Researchers are making great efforts to identify patients who may benefit from targeted arrhythmia prevention and treatment by digging into new clinical markers for SCD risk stratification in early-stage HCM (18).

### New clinical markers for SCD risk stratification in early-stage HCM

In HCM patients who have not undergone significant structural changes, life-threatening ventricular arrhythmia depends at least in part on the cellular mechanisms of cardiomyocyte dysfunction. The changes in ion channel and intracellular Ca2+ homeostasis play a key role here (19, 20), similar to the pathogenesis of “genetic ion channelopathy.” With the absence of a good genotype-phenotype correlation, specific genotypes are not recommended for SCD risk stratification in most cases. However, based on cellular models, animal models, and patient samples, studies on the arrhythmogenic mechanism of gene mutations and the subsequent molecular expression profile and functional analysis are invaluable for precise and individualized treatment in HCM.

Patients with sarcomere mutations are more prone to SCD (21). A study conducted on Japanese HCM patients found that carriers of the MYBPC3 mutation were associated with a higher incidence of ventricular arrhythmia and syncope (22). Several studies found that five mutations (MYH7-R403Q, MYH7-R453C, MYH7-G716R, MYH7-R719W, and TNNT2-R92W) were associated with a high incidence rate of SCD and, therefore, considered these mutations “high-risk” (13). Other studies also found that patients with two or more pathogenic sarcomere gene variants are more prone to fatal arrhythmias and a deteriorated clinical course and that the presence of multiple rare variants was associated with an increased risk of SCD (23). However, the incidence of SCD events in most HCM cohorts is relatively low because of the limited number of cases included (16). Moreover, identified pathogenic sarcomere variants in HCM may be more the exception rather than the rule, and overall SCD incidence is low. Some large patient databases screening results showed that common polygenic modifiers more likely influence the SCD development in HCM (4).

## Cellular mechanism of ventricular arrhythmias in early-stage HCM

### Pros and Cons of the various research models

The leading experimental platforms of preclinical research include surgically harvested human samples, transgenic animal models, and patient-derived induced pluripotent stem cells (hiPSCs).

#### Human myocardial specimens

Human myocardial samples show a high translational value. Freshly isolated human cardiomyocytes were quickly detected *via* the patch-clamp technique. For electrophysiological research, such as changes in the late sodium current (INaL), L-type calcium channel current (ILTCC), delayed rectifier potassium current, etc. Separated single-muscle trabeculae could be utilized for mechanical properties and isometric contraction studies (24). Limitations such as sample scarcity and wide heterogeneity exist in human samples research (genetic variation, various clinical manifestations, and environmental effects). In addition, patients who underwent surgical resection of the hypertrophic ventricular septum are representative of late-stage HCM (24); so, it is difficult to determine whether the observed electrophysiological or calcium homeostasis changes need to be attributed to the direct effect of the gene mutation or the secondary changes caused by myocardial remodeling and disease progression.

#### Transgenic animal models

Animal models are a crucial research platform for studying the pathophysiology, disease progression, and also for testing new therapies for HCM. Inbred lines eliminate genetic background variability and help deeply understand the pathogenic role of genetic variation in HCM and downstream pathogenic signaling pathways. The study of the pathological changes at different progression stages of HCM provides outstanding support for verifying potential interventions. Rodent models, such as the α-MyHC R403Q mouse that corresponds to the human β-MyHC R403Q mutation, showed myocyte disorders, interstitial fibrosis, and diastolic dysfunction, effectively replicating the pathological changes observed in humans, along with the significantly increased incidence of arrhythmia (25). The rodent model has a relatively short effective refractory period (ERP) due to its fast heart rate, limiting its use in assessing the risk of ventricular arrhythmia, an additional reason why rodents are not good models for studying ventricular arrhythmogenesis is that their hearts are too small to generate sustained reentry-based arrhythmias spontaneously. Other animal models include rabbits (suitable for echocardiography and invasive electrophysiological research), zebrafish (easy for gene editing and high-resolution *in vivo* cardiac imaging), and large animal models such as cats and pigs. Transgenic pigs have emerged as a potential ideal model to study arrhythmia *in vivo*, considering their similarity with humans in terms of the genotype, phenotype, cardiac anatomy, and electrophysiology. However, the gap between animals and humans in ion channel components, cardiac electrophysiology, and the cardiac structure are the drawbacks of HCM research based on animal models. Also, in transgenic animal models, it is difficult to distinguish the primary abnormal effects directly caused by gene mutation defects from the secondary compensatory changes in molecular and physiological remodeling during the pathogenesis of HCM (26).

#### Patient-derived induced pluripotent stem cells

The hiPSCs have triggered a revolution in basic and clinical research and currently play a leading role in the study of the pathological mechanism of hereditary heart diseases such as HCM. The innovative tool of hiPSCs overcomes the limitations of using human surgical samples and transgenic animal models (27). Its main advantages are an adequate material source, controllable directional differentiation, a complete replication of the patient's gene map, suitability for preclinical drug development, and safety toxicology screening (27). More importantly, genome-editing technologies such as Clustered Regularly Interspaced Short Palindromic Repeat (CRISPR)/Cas9 and Transcription Activator-like Effector Nuclease (TALENs) would quickly generate knockout and knockin or report cell lines and control cell lines with the same genetic background. Isogenic control enables researchers to directly associate genetic defects with any observed phenotypic variation (28, 29). For example, the calcium indicator gene can be knocked in using CRISPR/Cas9 technology to generate the calcium reporter iPSC-CM cell line. Therefore, a confocal line-scanning analysis directly shows the arrhythmia-related calcium signal, and different sarcomere gene mutations lead to inconsistent contractility and molecular expression spectra, suggesting the necessity of specific treatment (28, 30).

CRISPR/Cas9 could also be utilized to create a set of functional analysis platforms in iPSC-CM. RNA sequencing, transcriptome analyses, and myofilament calcium sensitivity work together to explore the pathophysiology of TNNT2 variation, which is helpful in determining the SCD risk and early interventions (31). In addition, machine learning also helps analyze the calcium transient signal parameters of iPSC-CM and clarify the differential diagnosis of HCM (32).

Despite this, iPSC-CM has some defects, such as immature cell differentiation and a status similar to that of fetal cardiomyocytes in electrophysiology, electromechanical properties, and morphology. The critical and unanswered question is whether (immature) iPSC-CM really reflect the phenotype of the patient or predict it (33). In the recent work of Han et al., the repolarization in iPSC-CM has little resemblance with that of mature undiseased ventricular myocytes (27). The intrinsic immaturity of intracellular Ca2+ handling mechanism possibly explained the reason that the iPSC-CMs recapitulate poorly fundamental physiological properties of adult CMs, according to a novel *in silico* model of iPSC-CMs and mathematical platform (34). Specifically hiPSC-CM do not necessarily recapitulate findings from transgenic animals bearing classical mutations in HCM (35). At present, promising strategies to improve the maturity of hiPSC-CM, such as long-term culture on nanopatterned surfaces, 3D culture systems, and other engineered heart tissue (EHT) methods (36), have emerged.

### Ion channel remodeling: A possible arrhythmogenic mechanism

The results of the patch-clamp study based on myocytes isolated from HCM patients showed that compared with cardiomyocytes from patients without heart failure and cardiac hypertrophy, ILTCC and INaL were significantly increased in HCM cardiomyocytes. However, the inward rectifier K+ current (Kir2.1/IK1), transient outward K+ current (Kv4.3/Ito) and delayed rectifier K+ current (IKs, IKr) were significantly reduced (24). However, it is not clear whether it directly results from a gene mutation. Studies have found that TNNT R92Q-mutant mice develop a complete HCM phenotype at approximately 6 months; however, four-week-old mice already demonstrate markedly increased INaL before hypertrophy and diastolic dysfunction phenotypes. Further rescue experiments also confirmed that the lifelong ranolazine treatment effectively prevented most of the above abnormalities in adult mice (increased INaL density, decreased K+ channel gene expression, slowed Ca2+ transient, aberrant diastolic Ca2+, and augmented spontaneous diastolic depolarization). The increase in INaL during the early stage of HCM is a driving factor for subsequent pathological changes, suggesting that ranolazine can be used for prophylaxis in young HCM patients with high-risk gene mutations and a negative clinical phenotype (37). New progress has also been made in hiPSC-CM studies. Han et al. studied MYH7-R442G hiPSC-CM from HCM patients and showed most electrophysiological changes similar to those observed in adult human HCM myocytes, such as the prolongation of APD50 and APD90, Ca2+ current increase, INaL increase and K+ current abnormality (27). Furthermore, in engineered heart tissue (EHT) formed by a three-dimensional ACTN2 T247M hiPSC-CM cell culture, APD50 and APD90 were prolonged, the Ca2+ current increased, and the AP amplitude increased (38).

To further explain the association between various sarcomere mutations and ion channel remodeling in experimental models, the potential mechanistic connections point to the critical calcium regulatory protein calcium calmodulin-dependent protein kinase II (CaMKII), which was proved to bridge the gap between gene variants and arrhythmogenic ion channelchanges (39). The autophosphorylation of CaMKII enhances its activity on downstream targets such as the phosphorylation of LTCC β-subunits, slowing down current inactivation and prolonging the entry of Ca2+ during systole (40). The phosphorylation of the Na+ channel (Nav1.5) changed the inactivation of INa, increasing INaL. The enhanced INaL hinders the function of the sodium-calcium exchanger (NCX) and leads to the further accumulation of Ca2+ during diastole, which, in turn, leads to the further enhancement of CaMKII activity and forms feedback activation. Therefore, enhanced CaMKII activity can be considered the cause of action potential remodeling, representing the link between the initial cytosolic Ca2+ accumulation and early ion channel changes. In conclusion, augmented INaL and ILTCC are common in early-stage HCM, as confirmed in various HCM experimental models. The mechanism is generally the “posttranslational modification” of ion channels; therefore, it increases the occurrence of arrhythmogenic EAD and DAD (41).

### Possible arrhythmogenic mechanism: Enhanced myofilament Ca2+ sensitization

Troponin is a set of regulatory proteins composed of three domains: troponin I (cTnI), troponin C (cTnC), and troponin T (cTnT). They allow the interaction between myosin and actin to form cross-bridges after removing tropomyosin (TM) from actin filaments by binding with calcium.

Troponin mutations that increase myofilament calcium sensitivity are associated with familial HCM and lead to a high risk of SCD. Mice carrying the MYBPC3-targeted knock-in mutation (G>A transition on the last nucleotide of exon 6) exhibited higher myofilament Ca2+ sensitivity, faster Ca2+ transient decay, and diastolic dysfunction (42). HCM patients with the TNT-I79N mutation are susceptible to SCD even in the absence of extensive fibrosis or ventricular hypertrophy (43). Therefore, the study of the arrhythmogenic mechanism of the TNNT2 gene mutation is helpful for the early screening and intervention of SCD-susceptible asymptomatic HCM patients. The rescue of abnormal calcium sensitivity can delay or prevent the development of the HCM phenotype. After the Tm-E180G HCM mouse model was hybridized with low myofilament calcium sensitivity mice, the double transgenic mice showed a significant improvement in the HCM phenotype (44). However, a recent study of the arrhythmia mechanisms in Mybpc3-KI mice showed that increased myofilament Ca2+ sensitivity is not sufficient to induce arrhythmias in this mouse model and pointed to the reduced K+ currents as a pro-arrhythmic trigger in Hom-KI mice even at early disease stages. But neither human EHTs derived from CRISPR/Cas9-generated homozygous MYBPC3 mutant (MYBPC3hom) nor the left ventricular septum samples obtained from HCM patients indicated relevant reduction of K+ currents (35). Species differences complicated the translatability of rodent animal-based findings into human samples and human-like models.

#### Triangular AP morphology, shorted ERP and enhanced reentry circuit formation

Under low-frequency stimulation or steady-state, the positive calcium efflux mechanism of NCX promotes an inward current in the third phase of an action potential. The enhanced myosin calcium sensitivity slows diastolic calcium dissociation and reduces the Ca2+ transient amplitude and dynamics, reducing the NCX current and shortening early repolarization (APD70).

Using CRISPR–Cas9 technology, the I79N-TnT hiPSC-CM cell line derived from skin fibroblasts showed myofibril disorder and increased arrhythmogenic events, indicating the direct result of the I79N-TnT mutation on damaged cytosolic Ca2+ buffer and the decrease in the Ca2+ transient amplitude (45). Another study that used the human recombinant fine myofilament (hcRTF) model and heterozygous I79N(+/-) hiPSC-CMs showed that the TnT-I79N mutation could increase myofilament calcium sensitivity and calcium buffer and weaken calcium transients, leading to arrhythmogenic calcium alternation and AP morphology triangulation (43).

#### Increased diastolic Ca2+, sarcoplasmic reticulum (SR) Ca2+ overload, and the substrate for trigger activity

Under high-frequency stimulation, the enhanced cytosolic Ca2+ buffer will lead to the attenuation of Ca2+ transients and the accumulation of Ca2+ during diastole, promoting the spontaneous SR Ca2+ release, triggering activities such as EADs and DADs (46).

TnT-I79N cardiomyocytes showed slower Ca2+ transient kinetics and increased diastolic Ca2+ concentration (especially at a high stimulation rate), and the SR Ca2+ content also increased significantly, resulting in SR Ca2+ overload and spontaneous Ca2+ release events (47). Compared with the mouse HCM model, guinea pigs are more suitable for studying the effect of changes in myofilament Ca2+ affinity. Isolated guinea pig cardiomyocytes were transfected with the adenovirus, inducing the expression of TNT-R92Q, TNI-R145G, and Tm-D175N (48). The data showed that the introduced mutations significantly enhanced Ca2+ binding, resulting in higher Ca2+ buffer capacity, increased diastolic Ca2+ and CaMKII activation, and increased SR calcium leakage, suggesting that the change in the Ca2+ buffer in myofilaments is the main starting point of the signal cascade and that directly targeting the myofilament Ca2+ sensitivity may be an attractive treatment option for HCM. MYL2 R58Q is associated with severe cardiac hypertrophy and SCD risk. MYL2 R58Q hiPSC-CMs also found that the percentage of myofibrillar disordered and irregularly-beating cells was significantly higher than that of control cell lines, with a lower peak value of calcium transients, increased attenuation time, and compensatory reduction of the peak of ILTCC (49).

The increased cytosolic calcium and SR Ca2+ overload and posttranslational modification of the ryanodine receptor (RyR) [such as RyR hyperphosphorylation of the CaMKII sensitive site (50)] by activated CaMKII jointly promote the increase in the RyR opening probability, which has been confirmed in human specimens and a mouse HCM model (50, 51).

### Other possible arrhythmogenic mechanisms

The degree of myocardial ion channel remodeling is uneven in the whole heart, and areas of severe dysfunction and normal function without abnormal electrical activity coexist in different areas of the left ventricle. The HCM ventricle computer model reproduces the uneven distribution of myocyte electrophysiological changes, indicating that the increased dispersion of repolarization may be an essential factor for persistent ventricular arrhythmia in HCM (52).

The transverse tubules (TTs) in cardiomyocytes facilitate the rapid propagation of action potential signals to the interior of cardiomyocytes and trigger synchronous SR Ca2+ release (53). The destruction or redistribution of TTs impaired the synchronization of Ca2+ release, accompanied by the possibility of APD increase and ERP alternation, which promotes the formation of reentry circuits and constitutes one of the possible mechanisms of arrhythmogenic activity (54), confirmed in the Δ160E cTnT HCM mouse model (54).

In some genotype-negative HCM patients, mutations in calcium regulatory genes, such as RyR2-P1124L, may increase RyR2 calcium sensitivity and induce arrhythmia and secondary structural cardiac remodeling (55). In addition, RyR2 may interact directly with MYBPC3, affecting intracellular calcium homeostasis when its gene mutation or conformational change occurs (56).

The redox state could be another essential factor in the phenotype of familial HCM, as it reverses the overactivated oxidative stress, improves overactivated myofilament calcium sensitivity, and attenuates hypertrophy and diastolic dysfunction. The sphingosine-1-phosphate receptor regulator, FTY720, can also improve myofilament calcium hypersensitivity by improving the level of oxidative stress (57). Cardiomyocyte myeloperoxidase (MPO) inhibition alleviates the relaxation defect in hypertrophic iPSC-CMs through MYBPC3 phosphorylation, indicating a treatment strategy that can be readily investigated in the clinical setting (58).

## Drugs against ventricular arrhythmia in HCM

### INaL inhibitors

Increased INaL leads to APD prolongation, which increases the possibility of EAD and DAD, promoting calcium influx at the plateau phase, further aggravating calcium overload, and predisposing patients to arrhythmic diastolic dysfunction. INaL inhibitors shorten the APD and reduce calcium overload (20, 40). It was also proved in cardiomyocytes isolated from septal myectomies of HCM patients as INaL inhibitor Ranolazine showed beneficial effects by lowering intracellular Ca2+ levels (24). Besides, Ranolazine improved tolerance to high workload in isolated cardiomyocytes from Mybpc3-KI mice, not by blocking INaL, but by antagonizing β-adrenoceptors and slightly desensitizing myofilaments to Ca2+. Moreover, chronic ranolazine application in Mybpc3-KI mice did not have translatory effects in cardiac phenotype *in vivo* (59). The low selectivity and species discrepancy of Ranolazine hampers the interpretation of drug studies. RESTYLE-HCM (EudraCT2011-004507-20) and RHYME (NCT01721967) clinical trials have confirmed that the INaL inhibitor, ranolazine, could not improve the exercise ability. However, it significantly reduces the PVC frequency on 24-h ambulatory ECG, confirming its promising anti-arrhythmic ability.

In addition, Eleclazine (GS-6615) is a novel, selective INaL inhibitor and is more potent and selectively inhibiting compared with ranolazine. A placebo-controlled study (LIBERTY-HCM) involving 40 treatment centers carried out to evaluate the therapeutic effect of eleclazine on exercise endurance, diastolic function improvement, heart failure, arrhythmia, and other complications in HCM patients. Unfortunately, eleclazine (GS 6615) failed to show a reduction of ICD shocks and pacing events in patients with VT and VF when compared to a placebo, and the concurrent studies looking at safety and efficacy of the drug in HCM and Long-QT syndrome type 3 patients populations were both halted (60).

Besides, novel selective INaL inhibitor, GS-967, has been shown to abolish arrhythmias induced by isoprenaline in cardiomyocytes and trabeculae from HCM patients, therefore GS-967 have the potential to ameliorate symptoms caused by inducible obstruction in HCM patients (61). However, the selectivity inhibitory effect of GS-967 has been questioned recently in canine ventricular myocardium model and was suggested to be classified as a class I/B antiarrhythmic agent (62).

As a sodium channel blocker and second-line therapeutic option for HCM, disopyramide was also found to stabilize the closed state of the RyR2 channel. In addition, it can inhibit Ca2+ dependent arrhythmias and DADs by reducing cytosolic calcium levels (63).

### Calcium homeostasis regulatory agents

Three significant changes in calcium homeostasis related to sarcomere gene mutations are the increase in myosin calcium sensitivity, the decrease in SR calcium reuptake, and cytosolic calcium overload. Disorders of calcium homeostasis aggravate myocardial hypertrophy, fibrosis, and diastolic dysfunction and increase the incidence of arrhythmias and sudden cardiac death (46).

The myosin calcium inhibitor, blebbistatin, reduces myocardial calcium sensitivity by inhibiting the formation of the transverse bridge and the interaction between myosin and actin, as a direct non-selective myosin inhibitor, blebbistatin was tested on HCM models *in vitro* for research purposes only. The TnT mutation in the HCM animal model has proven its antiarrhythmic effect, which can provide potential targets for future gene therapy (64), but relevant clinical trials have not yet been carried out.

Mavacamten, also known as MYK-461, is a selective allosteric inhibitor of myosin ATPase that can specifically inhibit the overactivation of ATPase caused *via* the myosin MYH7 mutation, reversibly inhibit the myosin and actin coupling reaction, slow down the cross-bridge cycle, and reduce the calcium sensitivity of myocardial myosin, resulting in a decrease in the sarcomere contraction fraction and myocardial contractility. In a transgenic mouse HCM model, MYK-461 prevented the development of left ventricular hypertrophy and fibrosis (65, 66). In iPSC-CMs, MYK-461 effectively improved the percentage of myosin heads in the overrelaxation state (67). The drug has been proven to be effective and safe in patients with obstructive or non-obstructive HCM through the PIONEER-HCM study (NCT02842242), the EXPLORER-HCM study (NCT03470545) (68), and the MAVERICK-HCM study (NCT03442764). The ongoing VALOR-HCM study (NCT04349072) has also achieved satisfactory results recently; thus, the drug is expected to become the first myocardial myosin inhibitor for the treatment of HCM. Its preventive effect on ventricular arrhythmia and SCD has not been discussed in detail; however, considering the improved functional status, clinical symptoms, and quality of life, its effectiveness in inhibiting arrhythmia and SCD is worthy of special attention.

In a variety of HCM models, it was found that abnormal sodium channel and calcium channel currents preceded structural changes such as myocardial hypertrophy or fibrosis, and early medication against ion remodeling can effectively correct the HCM phenotype, suggesting the importance of preventive medication and long-term medication strategies.

## Summary and conclusions

HCM, the most common inherited heart disease, is associated with an increased risk of ventricular arrhythmias and SCD. Young and asymptomatic patients, including professional athletes, are not immune to this risk. ICDs effectively terminate the group's malignant ventricular arrhythmias; however, they are also associated with severe incidence rates, such as inappropriate electrical shocks and device-related complications. Therefore, it is imperative to guide the accurate prediction of ICD implantation using the SCD risk stratification system. Over the past few decades, the updated SCD scoring system, recommended separately by the AHA and the ESC, has made real-world ICD application decisions more complicated, highlighting the need to refine the risk stratification system further.

Since the electrophysiology related to HCM is similar to the pathogenesis of ion channelopathy, it is necessary to apply the management experience of ion channelopathy patients in future clinical practice in HCM.

Individualized risk prediction is expected to achieve the most accurate risk assessment and reasonable interventions. Deciphering the genetic basis of HCM makes routine gene detection possible and partially clarifies the primary mechanism of HCM. The yield of gene testing is expected to increase in the next few years, and the possibly so-called missing causal genes or unknown SNP variants will be identified. According to those large patient databases screening results in the past few years, it is not certain that many more single-gene causes of HCM will be identified, conversely, it is more likely that polygenic modifiers will be found to influence the SCD development in HCM. These advances are also expected to enable the development of additional specific therapies and the editing of mutations in HCM.

Given the diversity of the mechanisms involved, iPSC-CM derived from specific patients could be used for translational investigations of electrophysiological changes, abnormal calcium homeostasis, and structural matrix remodeling. Whole-heart computerized simulations with anatomical correction based on individual MRI and high-resolution ECG data, combined with gene therapy approaches, make the likelihood of SCD prevention a reality and fulfill the aspiration of reduced mortality for vulnerable HCM patients.

## Author contributions

HS and RW contributed to the conception and design of the work. HS, S-YD, and M-SR contributed to the acquisition, analysis, and interpretation of data for the work. All authors collectively drafted and critically revised the manuscript, gave their final approval, and agree to be accountable for all aspects of the work ensuring integrity and accuracy.

## Funding

This work was supported by a grant from the Innovation and Cultivation Programs Foundation of The Sixth Medical Center, Chinese PLA General Hospital (No. CXPY202117).

## Conflict of interest

The authors declare that the research was conducted in the absence of any commercial or financial relationships that could be construed as a potential conflict of interest.

## Publisher's note

All claims expressed in this article are solely those of the authors and do not necessarily represent those of their affiliated organizations, or those of the publisher, the editors and the reviewers. Any product that may be evaluated in this article, or claim that may be made by its manufacturer, is not guaranteed or endorsed by the publisher.
